# Vulva choriocarcinoma

**DOI:** 10.11604/pamj.2016.25.225.10462

**Published:** 2016-12-07

**Authors:** Houssine Boufettal, Naïma Samouh

**Affiliations:** 1 Centre Hospitalier Universitaire Ibn Rochd, Faculté de Médecine et Pharmacie, Hassan II Université de Casablanca, Casablanca, Maroc

**Keywords:** Choricarcinoma, vulva, trophoblastic neoplasia, chemotherapy, fertility

## Image in medicine

A patient aged 23 presented with a history spontaneous miscarriage which occurred five months earlier consulted for vulvar mass, painful, which gradually increased in size. On examination, the mass was sitting at the large right lip, it was inflammatory, firm and tender to palpation and measured five centimeters in diameter. Pelvic ultrasound was normal. Beta-h-CG (human chorionic gonadotrophin) quantitative plasma were highly increased to 562 000 IU / ml. The staging featuring a thoraco-abdominopelvic CT scan, chest X-ray and ultrasound abdomen and pelvis was normal. The biopsy of the mass objectified choriocarcinoma to vulvar seat. A methotrexate-based agent chemotherapy was introduced. The evolution was marked by the gradual decline of the mass until it disappears in four months. Plasma beta-h-CG had regressed and were normalized after three months of treatment. The outcome was favorable. With a decline of 24 months, no recurrence was noted.

**Figure 1:**
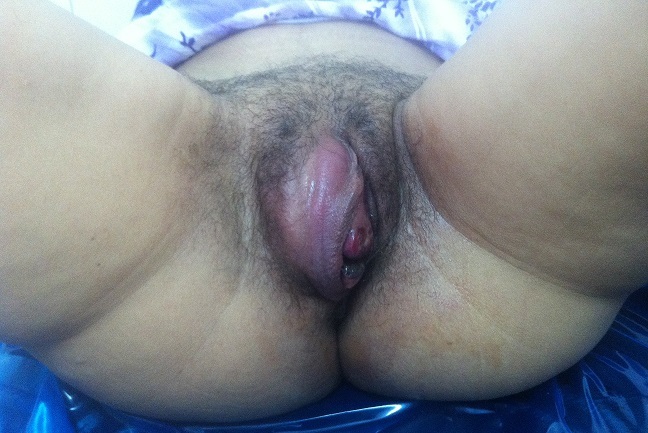
Mass was sitting at the large right lip, it was inflammatory, firm measured five centimeters in diameter

